# Plasmon enhanced light–matter interaction of rice-like nanorods by a cube-plate nanocavity

**DOI:** 10.1039/d1na00777g

**Published:** 2022-01-11

**Authors:** Hui Zhang, Huan Chen, Tingting Zhang, Xiaohu Mi, Zihe Jiang, Ziming Zhou, Lei Guo, Min Zhang, Zhenglong Zhang, Ning Liu, Hongxing Xu

**Affiliations:** Department of Physics and Bernal Institute, University of Limerick Ireland ning.Liu@ul.ie; School of Physics and Information Technology, Shaanxi Normal University Xi'an China zlzhang@snnu.edu.cn; Department of Electrical and Electronic Engineering, Southern University of Science and Technology Shenzhen China; School of Physics and Technology, Wuhan University Wuhan China hxxu@whu.edu.cn

## Abstract

Plasmonic nanocavity is widely used for enhancing light–matter interaction. Here, an efficient plasmonic nanocavity of the cube-plate system is constructed for the fluorescence enhancement of rice-like CdSe/CdS nanorods (NRs) with tunable emission wavelength. Over ten thousand times fluorescence enhancement is achieved with the assistance of the plasmonic nanocavity. Additionally, a small splitting effect is observed in both photoluminescence and scattering spectra of the NRs in the nanocavity owing to the intermediate coupling effect between the NRs and plasmonic nanocavity, which provides a potential application for optical signal enhancement and strong light–matter interaction.

## Abstract 2

Over 10 000 times fluorescence enhancement is achieved and small splitting is observed in both photoluminescence and scattering spectra of the NRs in the plasmonic nanocavity.

## Graphical abstract

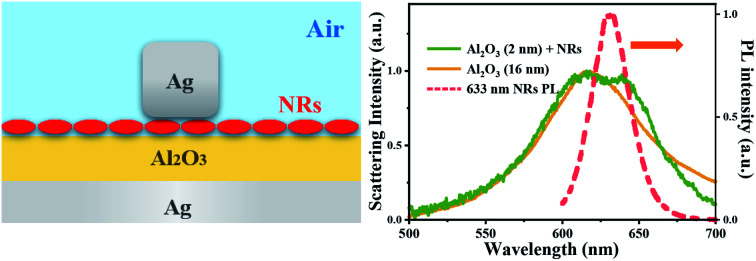

## Introduction

Plasmonic nanostructures have been one of the most promising solutions for enhancing the light–matter interaction due to their outstanding ability to confine light in a subwavelength range.^1–4^ Combining the advantages of the Purcell effect with nanoscale optical manipulation, plasmonic nanocavity systems perform extremely well in terms of generating significantly enhanced electromagnetic field in the nanogap.^5–7^ In addition, the wavelength of the surface plasmon resonance (SPR) can be easily tuned from ultraviolet to the infrared range by altering the geometric parameter of the nanostructure, which can be used to further enhance the light–matter interaction.^8–11^ Up to now, plasmonic nanocavity systems have been widely used for the applications of surface-enhanced Raman spectroscopy (SERS),^12,13^ surface-enhanced fluorescence (SEF),^14,15^ optical nonlinearity enhancement^16,17^ and intrinsic mechanism exploration of the light–matter interaction at the nanoscale.^18–21^

Quantum emitters, such as molecules, quantum dots (QDs), and semiconductor quantum wells, usually have a weak coupling efficiency with light in free space due to the mismatch of the length scale.^22^ The strong light confinement in the nanogap region of the plasmonic nanocavity systems provides a convenient platform for solving the length scale mismatch, which results in a series of optical signal changes depending on the interaction strength between the quantum emitter and the plasmonic nanocavity. Semiconductor QDs are popular light sources in device applications due to the advantages of tunable emission wavelengths at room temperature, excellent optical and chemical stability, and ease of integration with various types of substrates.^23–25^ However, while QDs are promising materials for the next generation of single-photon sources, their emission efficiencies need to be improved for a wider range of applications.

Herein, a silver nanocube-microplate-based plasmonic nanocavity system is proposed to enhance CdSe/CdS NR emission efficiency. The emission wavelength range of the synthesized CdSe/CdS NRs can be flexibly tuned by controlling the growth reaction time of CdSe. By regulating the Al_2_O_3_ spacer thickness between the nanocube and the microplate of the nanocavity, the SPR peak is tuned to match the emission wavelength of the NRs. Owing to the field enhancement and antenna effect, the fluorescence of the NRs is enhanced by more than ten thousand times. Furthermore, a small spectral splitting appears at the tip of the fluorescence and scattering peaks, which is attributed to the intermediate coupling between the NRs and plasmonic nanocavity. The near- and far-field electromagnetic properties are discussed in detail for the enhancement mechanism that is illustrated through the finite element method. This work sheds light on potential application for light–matter interaction enhancement.

## Methods

### Chemical synthesis

The synthetic procedure was based on the procedure reported in the literature.^26,27^ Tri-*n*-octylphosphine oxide (TOPO, 99%), tri-*n*-octylphosphine (TOP, 97%), tributylphosphine (TBP, 97%), *n*-octadecylphosphonic acid (ODPA, 97%), *n*-tetradecylphosphonic acid (TDPA, 97%), and *n*-hexylphosphonic acid (HPA, 97%) were purchased from Strem Chemicals. Cadmium oxide (CdO, 99.99%), sulfur (S, 99.98%), and selenium (Se, 99.99%) were purchased from Sigma-Aldrich. Typically, TOPO (1.5 g), ODPA (0.140 g), and CdO (0.030 g) were mixed in a 50 mL flask, heated to 150 °C, and alternately exposed to vacuum and argon at least five times until CdO was a brown solid and the rest of the reagents were a colorless liquid. Then, to dissolve CdO, the solution was heated to greater than 300 °C under argon until it became optically clear and colorless, which indicated that the reaction between CdO and ODPA was complete. Then, the temperature was increased to 370 °C, and 1.5 mL of TOP was injected into the flask, which caused the temperature to naturally decrease to 300 °C. Then, a Se/TOP solution (0.4 mL, 1 mol L^−1^) was injected at 380 °C and reacted for several minutes. The size of the CdSe core could be adjusted by altering the temperature, reaction time, and type of phosphonic acid (ODPA or TDPA). In a typical synthesis of CdSe/CdS nanorods *via* seeded growth, CdO (30 mg) was mixed in a 50 mL flask together with TOPO (1 g), ODPA (100 mg), and HPA (30 mg). After the flask was alternately exposed to vacuum and argon at least five times at 150 °C, the resulting solution was heated to 300 °C to become an utterly transparent liquid without any solids. Then, the temperature was increased to 350 °C and a mixed solution of S/TOP (0.5 mL, 2.5 mol L^−1^) and the above CdSe–TBP solution (100 μL) were injected into the flask, which caused the temperature to naturally decrease to 300 °C. The CdSe/CdS NRs were allowed to grow for approximately 8 min after the injection. Finally, the reaction mixture was cooled to room temperature, and an extraction procedure was used to separate the NCs from the side products and unreacted precursors. CdSe/CdS NRs with different length to diameter ratios were synthesized by adjusting the size of the core and the proportion of the shell precursor. Ag monocrystals were synthesized by a wet chemical method. In short, 1 mol of metal and hydrazine mixture was dissolved in 10 mL of deionized water with magnetic stirring. Subsequently, 10 mL of a 0.03 mol AgNO_3_ solution was added. Then the microplates were washed using ethanol and deionized water four times, and the silver nanocubes were purchased from Nanoseeds.

### Characterization and measurements

The morphologies of the NRs and silver nanocubes were characterized by using a JEOL-JEM 2100F transmission electron microscope (TEM) operating at an acceleration voltage of 200 kV. The high angle annular dark-field scanning transmission electron microscopy (HAADF-STEM) images were obtained with a FEI Titan cubed Themis G2 300 microscope operating at 200 kV and equipped with a probe aberration corrector and monochromator. The morphologies of nanocavities were characterized by using a Bruker scanning electron microscope (SEM). Optical measurements were conducted with a homemade confocal fluorescence microscopy system. The samples were vertically excited by using a semiconductor CW laser of 532 nm, and the *in situ* fluorescence spectra were collected by using a spectrometer (SP2750i, PI). The optical images were collected by using a 100× objective (NA = 0.9, Olympus). Oblique light illumination dark-field spectroscopy was used to acquire the scattering spectra of individual nanocavities. White light from a halogen lamp (50 mW, Olympus) was obliquely illuminated on the sample and an Olympus objective (50×, NA = 0.9) was utilized to collect the scattered light from the sample, and then the scattering spectral measurements were conducted with a LabRam HR evolution Raman system (Jobin-Yvon).

### Simulation

The numerical simulation of the near- and far-field distribution was carried out through the finite element method (FEM) using COMSOL Multiphysics software. 633 nm incident light enters through the airside into the nanostructure with the polarization direction along the *xy* plane of the nanocube. Sizes of the nanocavity systems are set corresponding to the results shown in Fig. 3a.

## Results and discussion

**Fig. 1:**
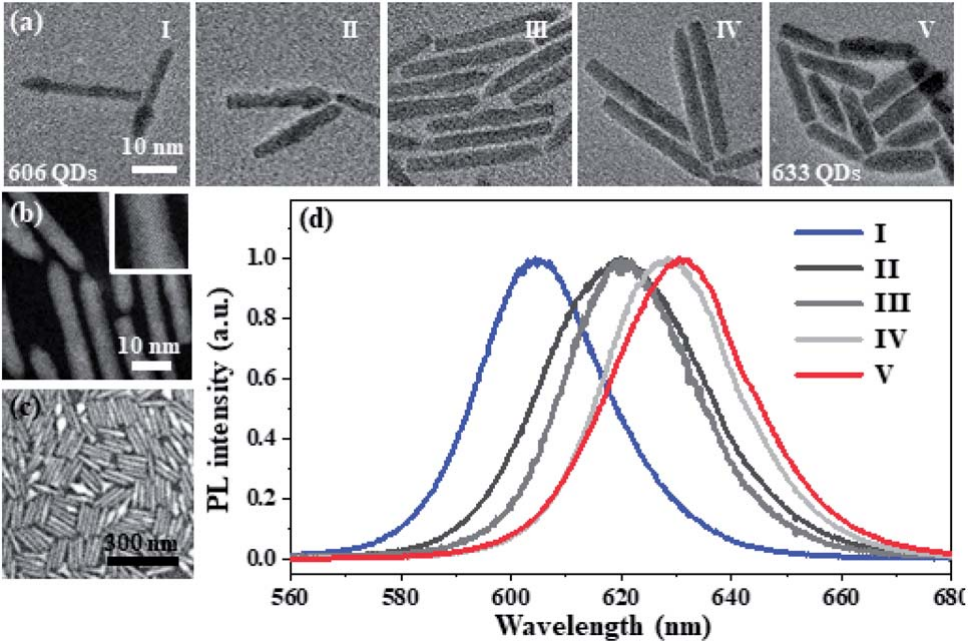
The tunable optical properties of CdSe/CdS NRs. (a) The transmission electron microscopy (TEM) image of the CdSe/CdS NR structures with different CdSe core reaction times, where the reaction time is 5 s (I), 10 s (II), 20 s (III), 30 s (IV), and 40 s (V), respectively. (b) The high-angle annular darkfield scanning transmission electron microscopy (HAADF-STEM) image of CdSe/CdS NRs with the reaction time of 5 s. The inset shows the HAADF-STEM image of a selected single CdSe/CdS nanoparticle. (c) The SEM image of the single-layer CdSe/CdS NRs on a silicon substrate. (d) The PL emission spectra of those CdSe/CdS NRs in Fig. 1a, where the emission wavelengths are 606 nm (I), 620 nm (II), 621 nm (III), 629 nm (IV), and 633 nm (V), respectively.

The optical properties of CdSe/CdS NRs can be tuned by controlling the growth process of CdSe. Fig. 1a shows the TEM images of the wet chemistry method synthesized CdSe/CdS NR structures with different reaction times. The CdSe core can gradually increase *via* improving the chemical reaction periods from 5 s to 40 s, which results in the quantum confinement effect change of the NRs. With the assistance of HAADF-STEM, the crystalline structure of the 633 CdSe/CdS NRs can be illustrated. As shown in Fig. 1b, it can be observed that the CdS shell is well covered on the surface of CdSe with a high crystallization performance. The synthesized NRs can form a single compact layer film on the silver microplate or silicon substrate (Fig. 1c), whose distribution can be adjusted *via* changing the concentration of the NR solution or the spin rate of the spin coater. The photoluminescence (PL) spectra shown in Fig. 1d clearly reflect the quantum confinement effect adjusted by the CdSe size, where the bandgap of NRs is dependent on the particle size resulting in the emission wavelength change from 606 nm to 633 nm.

**Fig. 2:**
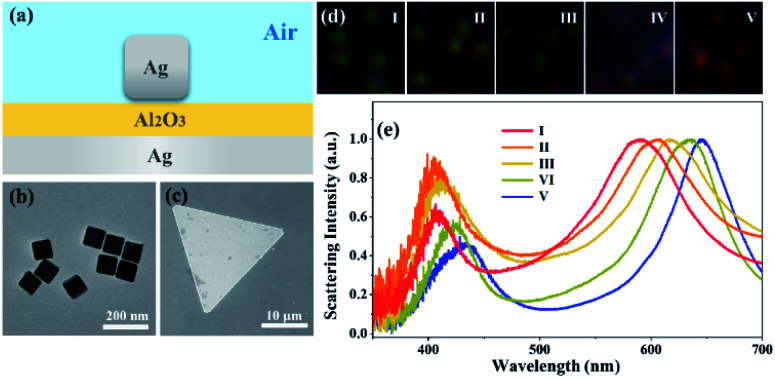
Tunable optical properties of the nanocavity system composed of the Ag nanocube, Al_2_O_3_ nanospacer and Ag microplate. (a) The cross-sectional schematic diagram of a single nanocavity system. (b) The TEM image of the synthesized silver nanocubes; the average size of the nanocubes is 82 nm. (c) SEM image of the nanocavity system, where the 82 nm Ag nanocubes are arbitrarily distributed on the 18 nm Al_2_O_3_ covered silver microplate. (d) Darkfield scattering image of the nanocavity system with different thicknesses of the Al_2_O_3_ nanospacer, where I is 26 nm, II is 18 nm, III is 16 nm, IV is 12 nm and V is 8 nm. (e) The scattering spectra of the single nanocavity system with different thicknesses of the Al_2_O_3_ nanospacer corresponding to (d).

To develop an efficient nanocavity system, the wet chemically synthesized monocrystalline silver nanocube and microplate are employed for plasmonic nanocavity due to the advantage of the atomic smooth surface of the microplate. As shown in Fig. 2a, the Al_2_O_3_ layer is used for adjusting the spacer thickness between the nanocube and microplate. Herein, the nanocubes with an average length of 82 nm (Fig. 2b) are deposited on the Al_2_O_3_ covered Ag microplate (Fig. 2c), where the thickness of the Al_2_O_3_ layer can be precisely controlled by the atomic layer deposition technique. The optical properties of the nanocavity system can be efficiently controlled by varying the distance between the Ag nanocube and microplate, which can be examined directly through dark-field images as well as detection using scattering spectra. Fig. 2d shows the dark-field scattering images of the nanocavity system with spacer thicknesses of 26 nm (I); 18 nm (II); 16 nm (III), 12 nm (IV) and 8 nm (V), respectively. With the Al_2_O_3_ thickness growth, the scattering color of the nanocavity gradually turns from red to green. The aforesaid event can also be observed in the scattering spectra, as illustrated in Fig. 2e, where two separate scattering peaks arise at 450 and 600 nm wavelengths, owing to the plasmonic hybridization effect between the silver nanocube and microplate. When the spacer thickness varies from 8 to 26 nm, the wavelength of the scattering peaks exhibits a distinct blue shift from 691 nm to 590 nm, which perfectly matches with the emission wavelength of the NRs mentioned in Fig. 1. As a result of integrating various NRs with various Al_2_O_3_ layer thicknesses, the above nanocavity system is suited for a comparatively wide range of emission wavelengths.

**Fig. 3:**
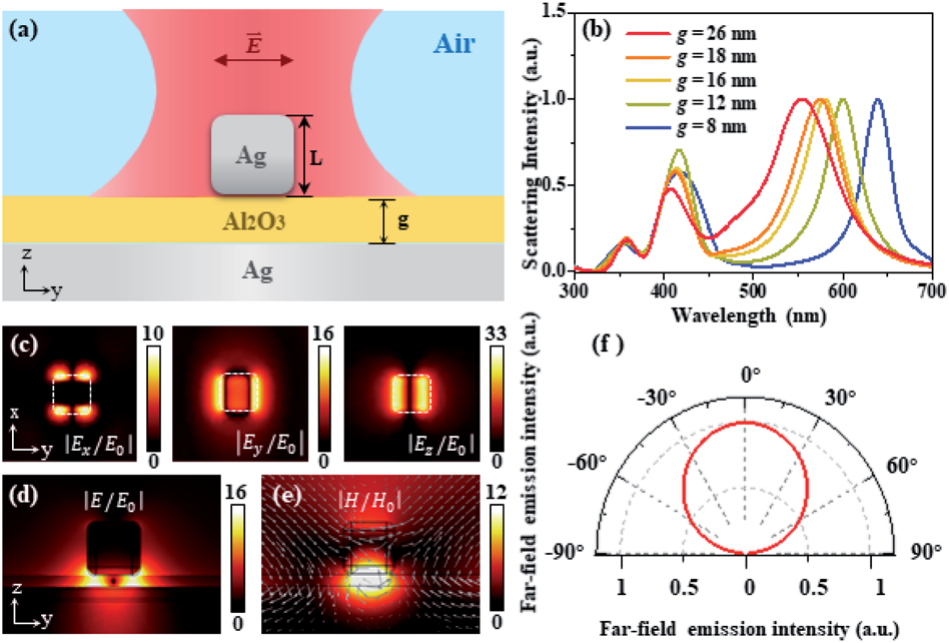
Simulation results of the nanocube based plasmonic nanocavity system. (a) Schematics and geometric parameters of the simulated plasmonic nanocavity system, where *L* represents the length of the nanocube, *g* is the thickness of the Al_2_O_3_ spacer, and the polarization direction of the incident light is along the *y*-axis. (b) Simulated normalized scattering spectrum with different thicknesses of the Al_2_O_3_ spacer. (c) Electric field distribution of the *x*, *y*, and *z*-components at the central plane of the Al_2_O_3_ spacer, which is normalized to the incident electric field |*E*_0_|. (d and e) Electric and magnetic field distributions of the nanocube based plasmonic nanocavity system, where the white arrow depicts the displacement currents. (f) The 2D angular far-field emission patterns in the *y*–*z* plane.

To further illustrate the performance and physical mechanisms of the nanocavity system, the finite element method (FEM) is used to simulate the electromagnetic properties of the nanocavity. Fig. 3a shows the schematic diagram of specific geometric parameters of the nanocavity excitation, where the size of the cube is 82 nm, the thickness of the silver microplate layer is 150 nm, and the deposition thickness of the Al_2_O_3_ spacer was adjusted from 8 to 26 nm. The relative permittivity of silver followed the experimental results reported by Johnson and Christy.^28^ From the simulated scattering spectra as shown in Fig. 3b, it is obvious that with the spacer thickness growth, the scattering peaks of the nanocavity system exhibit a distinct blue shift which matches perfectly well with the experimental results shown in Fig. 2e. The peaks around the wavelength 400–450 nm are attributed to higher order waveguide modes, where the peak around the wavelength 500–600 nm shows the electric quadrupole plasmonic cavity mode, which can be recognized as a standing wave pattern due to the cavity plasmon interference. Fig. 3c shows the electric field of the *x*, *y*, and *z*-components of the mode at NRs' emission wavelength. For the nanocavity system with an 18 nm Al_2_O_3_ spacer, the electric field amplitudes of the *x*, *y*, and *z*-components are 10, 16, and 33, respectively, where the amplitudes are normalized to the incident electric field |*E*_0_|. Fig. 3d shows the electric field distribution of the nanocavity region; the strong electric field enhancement occurs within the nanogap, which enables the strong optical interaction of the NRs with the nanocavity system and the enhancement of the optical signal of the NRs. From the magnetic field and displacement current distribution shown in Fig. 3e, the reverse polarity surface charges between the nanocube and Au microplate effectively form a current loop, indicating the generation of the magnetic dipolar mode, which results in the strong magnetic field enhancement in the nanogap. The above plasmon-induced magnetic resonance will help the nanocavity improve the ability to absorb light, which contributes to enhancing the optical signal in the nanogap region.^29,30^ Moreover, the formed in-plane magnetic dipole mode not only increases the absorption of light but also leads to the emission pattern with a single lobe and broad emission angle range (Fig. 3f). The antenna effect of the nanocube considerably improves the collection efficiency of the optical signal. As a result of the aforementioned results, the nanocube based plasmonic nanocavity system provides an efficient platform for enhancing the light–matter interaction of the NRs with the plasmonic nanocavity system.

**Fig. 4:**
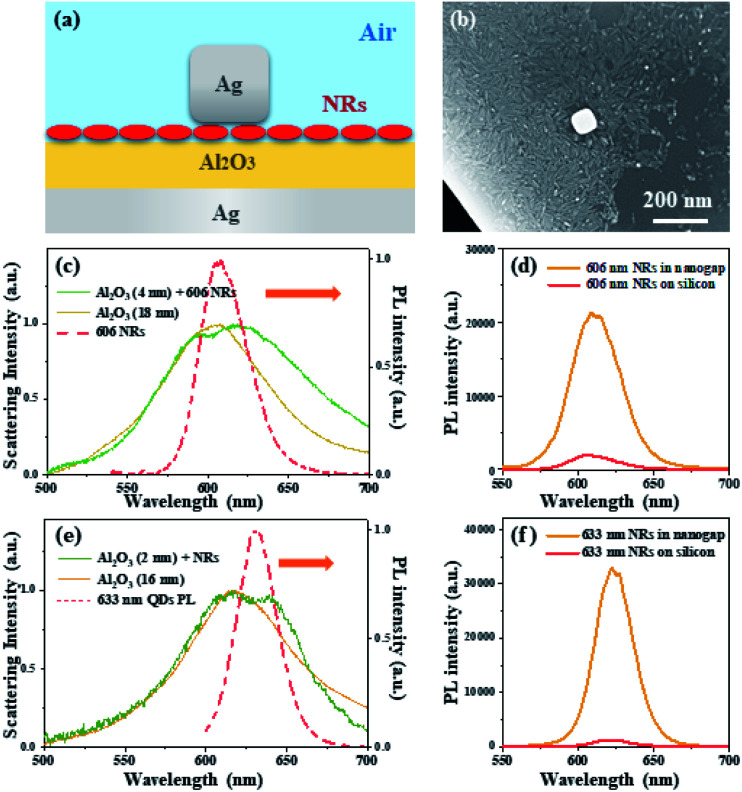
The optical properties of the nanocavity system composed of the Ag nanocube, NRs, Al_2_O_3_ nanospacer and Ag microplate. (a) The cross-sectional schematic diagram of a NR-nanocavity system. (b) The SEM image of the NR-nanocavity system, where a single 82 nm Ag nanocube is located on the silver microplate with single layer NRs and 2 nm Al_2_O_3_ covered. (c) The scattering spectra and photoluminescence (PL) spectrum of the single nanocavity system. The solid lines represent the scattering spectra with (solid green line) and without (solid yellow line) 606 NRs. The red dashed lines represent the PL spectrum of the 606 NRs inside the nanocavity system. (d) The PL spectra of the 606 NRs in the nanocavity and on a bare silicon substrate. (e) The single nanocavity system scattering spectra and photoluminescence (PL) spectrum. The solid lines represent the scattering spectra with (solid green line) and without (solid yellow line) 633 NRs. The red dashed lines represent the PL spectrum of the 633 NRs inside the nanocavity system. (f) The PL spectra of the 633 NRs in the nanocavity and on a bare silicon substrate.

The strong coupling effect between the nanocube and the microplate creates an ideal environment for the CdSe/CdS NRs to interact with the nanocavity system. The Al_2_O_3_ layer is employed to alter the cavity plasmon resonance peak to match the emission wavelength of the chosen NRs, as shown in Fig. 4a. Herein, the NRs with a 606 nm emission wavelength and Ag nanocube with a 82 nm length are selected for constructing the NR-nanocavity system (Fig. 4b). The Al_2_O_3_ layer with a 4 nm thickness is deposited on the surface of the silver microplate to obtain the 606 nm resonant wavelength consisting of the NR emission wavelength. As depicted in the solid green lines in Fig. 4c, the scattering spectrum of the NR-nanocavity system shows a distinct resonant peak at the wavelength of 606 nm. Comparing the scattering spectra of the nanocavity system with (solid green line) and without (solid yellow line) the NRs inside the nanogap, it is noted that the resonant peak of the NR-nanocavity system at 606 nm shows a noticeable broadening and a small splitting occurs. With the NRs inside the nanocavity, owing to the NRs arbitrarily distributed inside the nanocavity, the dielectric environment in the gap area exhibits an inconsistent spatial distribution. This non-homogeneous dielectric distribution will cause symmetry breaking between the TE and TM modes of the plasmonic nanocavity, resulting in broadening of the scattering spectrum.^23,31^ Additionally, coupling between the plasmon and exciton will also cause the scattering resonant peak broadening. The appearance of the small splitting of the scattering resonant peak supports the intermediate coupling effect between the NRs and the plasmonic nanocavity. From the scattering spectra shown in Fig. 4c, the NR-nanocavity coupling energy is apparently smaller than the linewidth of the cavity resonance, which proves that the mode splitting is driven by the intermediate coupling effect rather than the strong coupling effect.

The PL spectra presented in Fig. 4d exhibit the similar splitting due to the intermediate coupling effect. Compared with the emission properties of the NRs on a silicon substrate (solid red line), the NRs in the nanocavity (solid yellow line) display a significant enhancement and splitting of the fluorescence signal. Through the Purcell effect, the significantly enhanced electromagnetic field in the nanogap region will substantially improve the local density of optical states, resulting in a high spontaneous emission rate. Moreover, the signal collection efficiency can be improved due to the unidirectional upward radiation of the antenna effect of the nanocavity system. As a consequence of the Purcell-antenna effect synergy, the fluorescence intensity of NRs in the plasmonic nanocavities has been enhanced by approximately 6500 times when compared to NRs on a silicon substrate, as calculated using the PL enhancement factor (EF):^22^
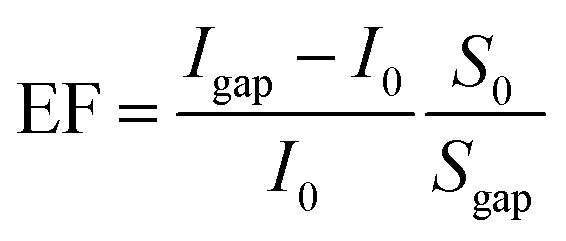
where *I*_gap_ is defined as the PL intensity from the nanocavity gap area with the silver nanocube and *I*_0_ is the PL intensity from the background NRs without the nanocube. *S*_0_ represents the area of the excitation laser spot with a diameter of 1.75 μm and *S*_gap_ is the area of within the hotspot around the nanocube.

Furthermore, the above behavior can also be observed by substituting alternative NR-nanocavity systems with different resonance wavelengths. Fig. 4e shows the scattering spectra of the nanocavity system with and without 633 NRs present inside the 16 nm nanogap, where the splitting of the resonant mode is much clear compared to the 18 nm NR-nanocavity system shown in Fig. 4c due to the stronger interaction between the NRs and plasmonic nanocavity. The weak splitting phenomenon occurring in scattering and PL spectra is mainly attributed to the following reasons. First, the broad resonant peak in scattering spectra indicates the comparatively significant loss of the plasmonic nanocavity system. Second, due to the mismatch between the electric field components and the nanorice-like NRs' dipole moment, the interaction strength between the NRs and the plasmonic nanocavity system will be considerably reduced. Third, the field enhancement and confinement are hampered by the comparatively thick spacer between the silver nanocube and the microplate. Although the NR-nanocavity system's splitting effects are very weak, the nanocavity system provides a solid foundation for fluorescence amplification. It is find that for the 633 NR fluorescence enhancement in the 16 nm NR-nanocavity system, the EF factor has been reached for over 10 000 times enhancement on the contrast with the one on the silicon substrate (Fig. 4f). Therefore, the NR-nanocavity system exhibits a great performance of signal enhancement, which provides an effective platform for exploring the plasmon–matter interaction mechanisms.

## Conclusions

In conclusion, a silver nanocube-microplate-based plasmonic nanocavity system is proposed to enhance CdSe/CdS NR emission efficiency. The center emission wavelength of the CdSe/CdS NRs can be flexibly tuned from 606 to 633 nm by controlling the growth reaction time of the CdSe core. Over 6500/10 000 times emission enhancement can be achieved for the NRs with the emission wavelength of 606 nm/633 nm. The plasmon resonance can be tuned from 580 to 700 nm to satisfy the emission wavelength of the NRs *via* regulating the Al_2_O_3_ spacer thickness of the nanocavity. It is noted that splitting is observed in both nanocavity scattering and NR fluorescence spectra due to the intermediate coupling between the NRs and plasmonic nanocavity. The enhancement and mode splitting mechanisms are also addressed, supported by electromagnetic characteristic simulations. This research could open up new avenues for optical signal amplification and light–matter interaction enhancement.

## Conflicts of interest

There are no conflicts to declare.

## Supplementary Material
