# Correction: Plasmon enhanced light–matter interaction of rice-like nanorods by a cube-plate nanocavity

**DOI:** 10.1039/d2na90021a

**Published:** 2022-04-21

**Authors:** Hui Zhang, Huan Chen, Tingting Zhang, Xiaohu Mi, Zihe Jiang, Ziming Zhou, Lei Guo, Min Zhang, Zhenglong Zhang, Ning Liu, Hongxing Xu

**Affiliations:** Department of Physics and Bernal Institute, University of Limerick Ireland ning.Liu@ul.ie; School of Physics and Information Technology, Shaanxi Normal University Xi’an China zlzhang@snnu.edu.cn; Department of Electrical and Electronic Engineering, Southern University of Science and Technology Shenzhen China; School of Physics and Technology, Wuhan University Wuhan China hxxu@whu.edu.cn

## Abstract

Correction for ‘Plasmon enhanced light–matter interaction of rice-like nanorods by a cube-plate nanocavity’ by Hui Zhang *et al.*, *Nanoscale Adv.*, 2022, **4**, 1145–1150, DOI: 10.1039/D1NA00777G.

The authors regret that an incorrect method was given for the synthesis of cadmium selenide/cadmium sulfide (CdSe/CdS) nanorods in the Chemical synthesis section.

The correct method is as follows:

In a typical synthesis of cadmium selenide/cadmium sulfide (CdSe/CdS) nanorods (NRs) *via* the seeded growth method, 78 mg cadmium oxide (CdO, 99.99%), 4.5 g trioctylphosphine oxide (TOPO, 99%) and 0.336 g tetradecylphosphonic acid (TDPA, 97%) were mixed in a 50 mL three-necked flask, heated to 150 °C, and alternately exposed to vacuum or argon gas for an hour. The solution was clear and colorless when the temperature was increased to 300 °C, which indicated the reaction between CdO and TDPA was complete. The temperature was then increased to 325 °C, and 2.25 mL trioctylphosphine (TOP, 97%) was injected into the flask. Subsequently, 0.95 mL Se/TOP stock solution (1 mol L^−1^) was quickly injected into the flask at 370 °C and maintained for 5 s, 10 s, 20 s, 30 s and 40 s, respectively. In CdS coating, a mixture of 57.9 mg CdO, 81 mg *n*-hexylphosphonic acid (HPA, 97%), 3 g TOPO and 0.3 g TDPA was heated to 150 °C and evacuated under vacuum or filled with argon gas alternately for 1 hour. 1.5 mL TOP was injected into the solution when the temperature was increased to 300 °C. The mixture of 1.5 mL S/TOP (2 mol L^−1^) and 0.425 mL synthesized CdSe core solution was injected into the flask quickly and maintained for 8 min at 320 °C. Subsequently, the solution was cooled to room temperature, purified and collected for further characterization. Ag monocrystals were synthesized by a wet chemical method. In short, 1 mol of metal and hydrazine mixture was dissolved in 10 mL of deionized water with magnetic stirring. Subsequently, 10 mL of a 0.03 mol AgNO_3_ solution was added. Then the microplates solution was washed by ethanol and deionized water four times, and the silver nanocubes were purchased from Nanoseeds.

The Royal Society of Chemistry apologises for these errors and any consequent inconvenience to authors and readers.

## Supplementary Material

